# Effect of frontal lobe lesions on the recollection and familiarity components of recognition memory

**DOI:** 10.1016/j.neuropsychologia.2008.07.003

**Published:** 2008-11

**Authors:** Sarah E. MacPherson, Marco Bozzali, Lisa Cipolotti, Raymond J. Dolan, Jeremy H. Rees, Tim Shallice

**Affiliations:** a Institute of Cognitive Neuroscience, University College London, London, UK; b Department of Neuropsychology, National Hospital for Neurology and Neurosurgery, London, UK; c Wellcome Department of Imaging Neuroscience, University College London, London, UK; d Dipartimento di Psicologia, University of Palermo, Italy; e Institute of Neurology, National Hospital for Neurology and Neurosurgery, London, UK; f SISSA, Trieste, Italy

**Keywords:** Recognition memory, Recollection, Familiarity, Frontal lobes

## Abstract

Single-process theories assume that familiarity is the sole influence on recognition memory with decisions being made as a continuous process. Dual-process theories claim that recognition involves both recollection and familiarity processes with recollection as a threshold process. Although, the frontal lobes of the brain play an important role in recognition memory, few studies have examined the effect of frontal lobe lesions on recollection and familiarity. In the current study, the nonverbal recognition memory of 24 patients with focal frontal lesions due to tumour or stroke was examined. Recollection and familiarity were estimated using the receiver operating characteristic (ROC) method. A secondary analysis was also conducted using standard signal detection theory methodology. Both analyses led to similar conclusions where only the familiarity component of recognition memory was impaired in frontal patients compared to healthy controls whilst the recollection-type (or variance ratio) processes remained intact.

There are two main theories to account for recognition memory: single-process (e.g. Dunn, 2004; Malmberg, Holden, & Shiffrin, 2004; McClelland & Chappell, 1998; Shiffrin & Steyvers, 1997) and dual-process theories (e.g. Jacoby & Dallas, 1981; Mandler, 1980; Tulving, 1985; Yonelinas, 1994). Both theories attempt to account for the subjective experience of familiarity and recollection. Familiarity is thought of as a feeling that an item has been presented but no additional information can be retrieved about the episode itself. Recollection involves remembering particular details about the experience when encountering an item.

According to the single-process theories of recognition memory, decisions are based upon an item's position along a single dimension (e.g. Dunn, 2004; Malmberg et al., 2004; McClelland & Chappell, 1998; Shiffrin & Steyvers, 1997). A signal detection view has been proposed where memory judgements are based on the comparison between the level on the dimension of the current item with a criterion. A recollection response is given when the memory strength of a test item goes beyond a high criterion. If the strength of a test item only surpasses a lower criterion, a familiarity response is given. Therefore, remember versus know judgments made by individuals reflect quantitatively different levels of confidence for the same memory trace or set of memory traces (e.g. Donaldson, 1996; Dunn, 2004; Malmberg et al., 2004; McClelland & Chappell, 1998; Shiffrin & Steyvers, 1997).

However, the dominant view in the literature, at least until very recently (Wixted, 2007), have been the dual-process theories which claim that recollection and familiarity are functionally independent component processes, both of which are important for judging whether an item has already been experienced (Jacoby & Dallas, 1981; Mandler, 1980; Tulving, 1985; Yonelinas, 1994). Some authors believe that the hippocampus and anterior thalamus support recollection while the surrounding temporal cortex, including the perirhinal cortex and the medial dorsal thalamus supports familiarity (Brown & Aggleton, 2001; Eldridge, Knowlton, Furmanski, Bookheimer, & Engel, 2000; Verfaellie & Keane, 2002; Yonelinas, Kroll, Dobbins, Lazzara, & Knight, 1998; Yonelinas, 2002). Others suggest that recollection and familiarity both depend on the hippocampus and perirhinal cortex (e.g. Manns, Hopkins, Reed, Kitchener, & Squire, 2003; Squire & Zola, 1997; Wixted & Squire, 2004).

The empirical data from patients with lesions in the medial temporal lobes are inconsistent as to whether recollection and familiarity are anatomically distinct. Some patients with bilateral hippocampal damage are reported to have selective deficits in recollection but not familiarity (MR: Bastin et al., 2004; YR: Mayes et al., 2004; KN: Aggleton et al., 2005). Certain group studies have also demonstrated recollection-specific deficits in patients held to have damage restricted to the hippocampus compared to patients with more extensive MTL lesions (Yonelinas, 2002; Turriziani, Fadda, Caltagirone, & Carlesimo, 2004). However, individual patients have also been reported who do not show sparing of familiarity following selective hippocampal damage. Patients with bilateral hippocampal lesions have been reported with significant reductions in both recollection- and familiarity-based recognition on tests using words and buildings as stimuli (JC: Bird, Shallice, & Cipolotti, 2007; VC: Cipolotti et al., 2006). In addition, patients with right hippocampal damage only have been shown to be impaired on both recollection and familiarity of topographical but not verbal and facial stimuli (RH: Bird et al., 2007). Also, in some group studies patients held to have bilateral hippocampal damage have been found to perform significantly more poorly than controls on both remember and know items using the remember/know procedure (Manns et al., 2003) and had lower recollection and familiarity estimates than controls using the ROC procedure (Wais, Wixted, Hopkins, & Squire, 2006; for reviews see Aggleton & Brown, 2006; Cipolotti & Bird, 2006).

Some memory models suggest that the frontal lobes also play an important role in recognition memory. However, it remains unclear whether the frontal lobes are involved in one or both of the recollection and familiarity processes. As the frontal lobes receive direct projections from the hippocampus and the medial portions of the thalamus, some authors argue that the frontal lobes are important for both components of recognition memory (Aggleton & Brown, 1999; Yonelinas et al., 2002). Others suggest that only recollection requires some additional processing by the frontal lobes (Davidson & Glisky, 2002; Knowlton & Squire, 1995; Tulving, 1989).

Most evidence for frontal lobe involvement has been provided by neuroimaging studies. Imaging data acquired during the encoding and retrieval phases of recognition memory has demonstrated that many regions of the prefrontal cortex are involved (for a review see Lepage, Ghaffar, Nyberg, & Tulving, 2000; Skinner & Fernandes, 2007). When contrasting brain areas associated with recollection and high confidence familiarity, Yonelinas, Otten, Shaw, & Rugg (2005) found recollection-related activation in the anterior medial frontal cortex (BA 10/32) and recognition confidence for non-recollected items positively correlated with left anterior (BA 10) and posterior prefrontal (BA 45/47) activation. More recently, Montaldi, Spencer, Roberts, and Mayes (2006) found activation in the bilateral inferior frontal gyrus (BA 47) and left medial frontal cortex (BA 10) when contrasting recollected scenes with strong familiarity. Increases in familiarity were associated with increases in the inferior frontal gyri and medial frontal cortex bilaterally. In a recent meta-analysis of the functional magnetic resonance imaging data, Skinner and Fernandes (2007) argued that both recollection and familiarity are related to right dorsolateral prefrontal activation, but recollection involves additional prefrontal activity in BA 6, 8 and 10. These results provide evidence for the involvement of the frontal regions in both recollection and familiarity, albeit different frontal regions.

The empirical data from patients with frontal lobe lesions are sparce. Earlier studies suggested that patients with focal frontal lesions do not demonstrate impairments in recognition memory (Janowsky, Shimamura, & Squire, 1989; Jetter, Poser, Freeman, & Markowitsch, 1986; Milner, Corsi, & Leonard, 1991; Stuss & Benson, 1984). More recently, studies have reported that recognition memory is affected, albeit with small effects. In particular, frontal patients can produce significantly higher false alarm rates on recognition memory tasks (Alexander, Stuss, & Fansabedian, 2003; Delbecq-Derouesne, Beauvois, & Shallice, 1990; Rapcsak, Polster, Glisky, & Comer, 1996; Rapcsak et al., 1998; Schacter, Curran, Gallucio, Milberg, & Bates, 1996; Swick & Knight, 1999). For example, in a study comparing recognition memory abilities of patients with frontal and patients with hippocampal lesions, Swick and Knight (1999) reported a double dissociation between the two patient groups. While the hit rates of the patients with unilateral frontal lesions were similar to controls, the patients produced significantly more false alarms. In contrast, while the hippocampal patients had a comparable false alarm rate to controls, their hit rate significantly declined as the retention interval increased. It has been suggested that this increase in false alarms reported in frontal patients is due to an over reliance on general characteristics common to both targets and distractors (Curran, Schacter, Norman, & Galluccio, 1997; Schacter et al., 1996). Therefore, distractors will appear familiar to frontal patients and they will report that the item has been presented before when in fact it has not. This would suggest that impaired recognition, in at least some frontal patients, may be due to deficits in familiarity rather than recollection processes.

To our knowledge, only one study in the neuropsychological literature has examined the effects of focal prefrontal lesions on recollection and familiarity (Duarte, Ranganath, & Knight, 2005). In this study, patients with unilateral frontal lesions performed only a recognition memory task employing meaningful objects. The stimuli were presented in either the left or right visual field and were considered as being contralesional or ipsilesional, depending on the patient's lesion site. Estimates of recollection and familiarity were obtained using the remember/know procedure. Frontal patients’ estimates of familiarity were significantly reduced compared to healthy controls but only when items were presented in the contralesional visual field. In contrast, estimates of recollection remained intact. This study suggests that the frontal lobes are critical for the familiarity, but not necessarily the recollection component of recognition memory.

Further indirect evidence showing a similar pattern of results was reported by Davidson, Anaki, Saint-Cyr, Chow, & Moscovitch (2006) who examined recollection and familiarity in Parkinson's disease (PD) patients. PD patients have been reported to perform similarly to focal frontal patients on tests of executive abilities and memory (see Bondi & Troster, 1997; McPherson & Cummings, 1996; Owen, 2004; Prull, Gabrieli, & Bunge, 2000; Taylor, Saint-Cyr, & Lang, 1990; Zgaljardic, Borod, Foldi, & Mattis, 2003). Using both the remember/know and process-dissociation procedure (PDP) procedures to estimate familiarity and recollection, Davidson et al. (2006) found a selective reduction in familiarity but not recollection in PD patients. These two studies suggest that it is the familiarity component rather than the recollection component of recognition memory that is impaired as a result of frontal lobe lesions.

The aim of the current study was to investigate within the context of the dual-process model further the contribution of recollection and familiarity processes in terms of nonverbal recognition memory in a group of patients with focal frontal lesions. According to previous neuropsychological data, we predicted that frontal patients would have reduced familiarity, but not recollection, estimates. The main difference between the current study and that of Duarte et al. (2005) is the method used to extract the recollection and familiarity estimates. Duarte et al. (2005) used the remember/know experimental paradigm to estimate recollection and familiarity. However, this procedure relies upon introspective processes where individuals must state whether they have a clear sense of re-experiencing the item or simply know that they have encountered it before. The primary purpose of analysis in the current study was to investigate the contribution of recollection and familiarity using the ROC method where hit rates are plotted against false alarm rates (Yonelinas, 1994). The dual-process model has been more recently adopted by the neuropsychological literature as it generates quantitative estimates of recollection and familiarity for patients and healthy controls. In turn, this allows for comparisons between the current data and other patient studies (e.g. Aggleton et al., 2005; Carlesimo et al., 2007; Cipolotti et al., 2006; Yonelinas et al., 2002).

There however still remains a debate as to whether one should adopt the dual-process model to describe ROCs in favour of rejecting the alternative unequal-variance signal detection (UVSD) model, which is compatible with a single-process view of recognition memory (Dunn, 2004; Malmberg et al., 2004; McClelland & Chappell, 1998; Shiffrin & Steyvers, 1997). Recently, based on a review of the literature, it was claimed that although both the dual-process model and UVSD fit the old-new recognition data well, the latter model fits it better (Wixted, 2007). Moreover, the UVSD procedure does not make any assumptions about the contribution of recollection and familiarity to recognition performance. Therefore, a secondary analysis was conducted using standard signal detection theory methodology. From z-ROC curves, the intercept provides a measure of sensitivity closely related to *d*′ and the slope provides a measure of the variance ratio between the strength of “old” items and “new” items (Macmillan & Creelman, 2003).

## 1 Material and methods

### 1.1 Patients

Twenty-four patients with frontal lobe lesions were recruited for the study from the National Hospital for Neurology and Neurosurgery. Patients were identified by a neurologist (MB) on the basis of MRI scans (or CT scans where MRI was unavailable). Only patients with lesions restricted to the frontal lobes were chosen. The scans were coded for the presence or absence of a lesion in the three main functional divisions of the frontal lobes in each hemisphere (lateral, medial and orbital). An area was only coded as damaged if at least 25% of that area was affected. The aetiologies were as follows: meningioma (*n* = 5), glioma (*n* = 14), haematoma (*n* = 1), anterior communicating artery aneurysm (*n* = 3) and traumatic brain injury (*n* = 1). Of the 19 tumour patients, 14 had undergone surgical excisions, including all five patients with meningiomas. Two of the glioma patients had undergone CT stereotaxic biopsies without excision. Frontal lesions were localised by operation site in the case of surgical patients or by gross tumour characterisation from T1-weighted images in the three non-surgical glioma patients. The mean time since surgery (excluding the three non-surgical glioma patients) was 17.91 months (S.D. = 20.68, range = 1–72). Five patients were left handed (four with right hemisphere lesions and one with a bilateral lesion). The mean age of the patient group was 44.96 years (S.D. = 14.84, range = 17–73) and the mean education was 13.79 years (S.D. = 2.93, range = 9–19). Table 1 shows the demographic information and lesion characterisation of the frontal patients.

### 1.2 Healthy controls

The performance of the frontal patients was compared with 33 healthy controls (16 men, 17 women). None of the controls had any previous history of head injury or stroke, major neurological or psychiatric illness, or alcohol abuse. Two were left handed. The mean age of the controls was 50.67 years (S.D. = 14.47, range = 17–72) and their mean education was 14.00 years (S.D. = 2.96, range = 10–19). The patients and the controls did not differ significantly in terms of age (*p* = 0.15) or education (*p* = 0.74). All participants were native English speakers. Consent was obtained according to the Declaration of Helsinki and the study was approved by the National Hospital for Neurology and Neurosurgery & Institute of Neurology Joint Research Ethics Committee.

### 1.3 MRI acquisition and analysis

Brain MRI scans for those 17 frontal patients without any contraindications that exclude them from MRI scanning were obtained at 1.5T (Siemens, Magnetom Sonata, Erlangen, Germany). In a single session, the following pulse sequences were collected from patients: (a) Fluid Attenuated Inversion Recovery (FLAIR) (TR = 10000 ms, TE = 96 ms, TI = 2600 ms); (b) 3D T1-weighted turbo-flash magnetization-prepared rapid-acquisition gradient echo (MPRAGE) (TR = 12.24 ms; TE = 3.56 ms, TI = 3530 ms, flip angle = 23°). For the FLAIR sequence, 52 contiguous interleaved axial slices were acquired with 3 mm slice thickness, with a 256 × 192 matrix over a 256 × 192 mm field of view, covering the whole brain. The 3D-MPRAGE sequence was acquired in a single slab, with a sagittal orientation, a 256 × 224 × 176 matrix size over a 256 × 224 × 176 mm^2^ field of view, with an effective slice thickness of 1 mm. FLAIR and T1-weighted images were coregistered using a linear affine algorithm with 12 degrees of freedom (SPM2, Wellcome Dept. Cogn. Neurol., London; http://www.fil.ion.ucl.ac.uk/spm). The frontal lesions were manually outlined on both, FLAIR and T1-weighted scans using MRIcro (http://www.sph.sc.edu/comd/rorden/mricro.html).

### 1.4 Data analysis

The performance of the frontal patients and healthy controls was compared using two-tailed independent samples *t*-tests unless otherwise stated.

### 1.5 Neuropsychological investigation

All participants performed a battery of standardised neuropsychological tests in order to assess intellectual function, memory and executive abilities. The National Adult Reading Test-Revised (NART) was administered to estimate premorbid levels of functioning (Nelson & Willison, 1991) and Raven's Progressive Matrices assessed nonverbal abstract reasoning (Raven, Raven, & Court, 1998). Memory functions were assessed with the Doors and People test (Baddeley, Emslie, & Nimmo-Smith, 1994). This is a standard neuropsychological test which allows the evaluation and comparison of verbal and visual recall and recognition memory. Verbal and visual recognition memory were assessed using the Names subtest and the Doors subtest, respectively. Verbal and visual recall memory were assessed using the People subtest and the Shapes subtest, respectively.

Measures of executive functioning were assessed: verbal fluency using Controlled Oral Word Association (letters F, A and S; Spreen & Strauss, 1998); set shifting using the Trail Making test (Reitan & Wolfson, 1985); and resistance to interference using the Stroop Test Colour-Word score (Trenarry, Crosson, Deboe, & Leber, 1989). Sustained attention was assessed using the Elevator subtest of the Test of Everyday Attention (Robertson, Ward, Ridgeway, & Nimmo-Smith, 1994) (see the individual sources cited for description of tests, procedures and norms). Concrete/abstract thinking was measured using a proverb interpretation test in which participants had to explain the meaning of eight common proverbs (e.g. Rome was not built in a day). For each proverb, participants were given 2 points for a fully accurate, abstract interpretation, 1 point for a partially accurate or concrete interpretation, and 0 for an inaccurate interpretation.

### 1.6 Experimental recognition memory test stimuli

The stimuli used were 120 black and white photographs of previously unknown buildings (e.g. Maguire & Cipolotti, 1998; Cipolotti & Maguire, 2003). The remainder were taken from the Photo Objects 50,000 Volume 2 CD-ROM (Hemera Technologies Inc., 2002). The photographs did not have any verbal cues (e.g. street names) or obvious distinguishing features present (e.g. people) to prevent the items being encoded in semantic memory. The photographs were presented in the middle of a computer screen using E-prime (2000).

### 1.7 Experimental recognition memory test procedure

The experimental ROC recognition memory test involved a study and test phase. During the study phase, the stimuli were presented at a rate of one every 3 s. Participants responded to each item according to whether they thought the building was attractive or not. These encoding instructions which are similar to those used in previous studies make the tasks more engaging for the participants and focus their attention on the presented stimuli.

The test phase, presented immediately after the study phase, was self-paced such that stimuli remained in front of the participant until a recognition judgement was made. The ROC procedure (Yonelinas, 1994, 2002) was adopted to achieve estimates of recollection and familiarity for frontal patients and controls. Judgements were made according to a 6-point confidence scale. If the item had already appeared in the study list (a target), participants should select confidence responses 4, 5 or 6:6 being the most confident and 4 being the least confident. If they thought that the item was new (a distractor), participants should select confidence responses 1, 2 or 3: 1 being the most confident and 3 being the least confident. Participants were told to make full use of the 6-point scale by spreading their responses across the entire range of confidence ratings.

### 1.8 Experimental data analyses

First the overall performance of the frontal patients and controls on the nonverbal recognition test was considered. The proportion of hits for targets (correct responses rated as 4, 5 and 6 on the confidence rating scale) and the proportion of correct rejections for distractors (correct responses rated as 1, 2 and 3 on the confidence rating scale) were calculated.

**Fig. 1:**
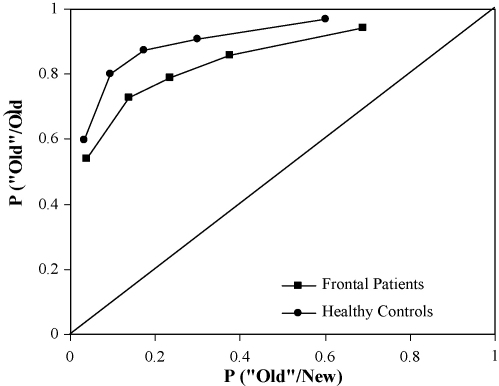
The average observed ROC for the frontal patients and the healthy controls.

A second analysis was conducted to examine the involvement of recollection and familiarity to the recognition judgments made by participants. Each participant's confidence ratings of their recognition responses were used to plot ROCs. The proportion of distractor (new) items rated as targets (old) was plotted on the *x*-axis and the proportion of targets (old) items rated as targets (old) was plotted against the *y*-axis. The first point on the function represents the proportion of hits and false alarms receiving the most confident 6 responses, and each successive point produces progressively more relaxed response criteria (i.e. items receiving 5 or 6 confidence responses, followed by items receiving confidence responses 4, 5 or 6 responses, and so on). Fig. 1 shows the average observed ROC points for the frontal patients versus controls.

The original Yonelinas et al. (1998) procedure was used to quantify recollection and familiarity. Recollection was estimated from the intersection of the regression line with the ordinate and at the same time familiarity was estimated by constraining the intercept with the *y*-axis in relation to the estimated probability that an item was recollected. A nonlinear equation was then fitted to the observed points of the ROC curve using a least-squares method through the solver in Excel (Yonelinas et al., 1998). In summary, while the estimate of recollection was taken as the intersection of the regression line with the ordinate for the most conservative response criteria, the degree of curvature was taken as an estimate of familiarity.

**Fig. 2:**
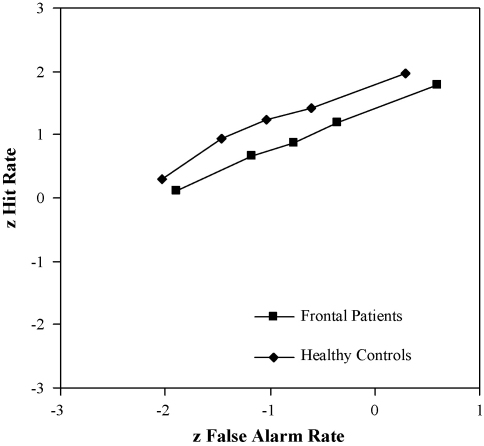
The z-transformed ROC data for the frontal patients and the healthy controls for buildings.

The current ROC data were also fitted to the UVSD model. In this model of recognition memory, the standard deviation of the target distribution is greater than the distractor distribution. When values could not be *z*-transformed because extreme confidence values were 1 for a “hit” or 0 for a “false alarm”, the probability was taken as midway between the obtained and the next possible score i.e. halfway between 59 and 60 out of 60 instead of 60 and halfway between 0 and 1 out of 60 instead of 0. Fig. 2 shows the ROC plotted in z-space to estimate the slope of the z-ROC and the *y* intercept value. Using this method to plot hit rates against false alarm rates, the single-process model of recognition memory predicts that the plotted z-ROC will be linear while the dual-process model of recognition memory predicts that the *z*-ROC will be slightly U-shaped (Slotnick, Klein, Dodson, & Shimamura, 2000; Wixted, 2007; Yonelinas, 1999a). One can estimate the variance ratio of the studied and unstudied items from the slope and the sensitivity (Da), which is calculated from the slope and the *y*-intercept value (see MacMillan & Creelman, 2003).

## 2 Results

### 2.1 MRI acquisition and analysis

Analysis of the MRI scans (or CT if MRI was not available) confirmed that all patients included in the study had lesions restricted to the frontal lobes of the brain. Ten patients had left frontal lesions (6 men, 4 women), 11 had right frontal lesions (5 men, 6 women) and 3 had bilateral frontal lesions (3 men). The scans were then coded for the presence or absence of a lesion in the lateral, medial and orbital divisions of the frontal lobes in each hemisphere. The coding procedure revealed that 13% had lateral only lesions, 21% had medial only lesions, 8% had orbital only lesions, 33% of patients had lesions involving the lateral and medial regions, 13% had orbital and medial lesions, 4% had orbital and lateral lesions and 8% had lesions extending into all three regions of the frontal lobes.

### 2.2 Neuropsychological findings

The frontal patients demonstrated a significantly lower level of premorbid functioning as assessed by the NART [106.17 versus 114.79; *t*(35.75) = −2.65; *p* < 0.05]. However, the frontal patients and controls did not differ significantly in terms of their Raven's Progressive Matrices scores [11.63 versus 12.82, respectively; *p* = 0.11].

Due to time restrictions, 1 patient and 4 controls were unable to complete the Doors and People test. The mean age-scaled scores and standard deviations for the frontal patients and controls assessed are reported in Table 2. The analysis revealed no recall/recognition dissociation as the frontal patients performed significantly more poorly than controls on all aspects of immediate memory [Names test: *t*(50) = −2.06; *p* < 0.05; Doors test: *t*(50) = −2.12; *p* < 0.05; People test: *t*(50) = −2.42; *p* < 0.05; Shapes test: *t*(50) = −2.07; *p* < 0.05]. The patients also performed significantly more poorly in terms of verbal forgetting [*t*(37.42) = −2.21; *p* < 0.05] but the two groups performed similarly in terms of visual forgetting (*p* = 0.20).

On the executive measures, the frontal patients produced significantly fewer words on verbal fluency [*t*(55) = −5.26; *p* < 0.0001], were significantly slower to complete the Colour-Word part of the Stroop Test [*t*(30.39) = 2.41; *p* < 0.05] and scored significantly more poorly on the Elevator subtest [*t*(23.66) = −2.32; *p* < 0.05] and proverb interpretation [*t*(39.60) = −2.81; *p* < 0.01]. However, the two groups did not significantly differ in terms of time to complete Part B of the Trail Making test (*p* = 0.15) (Table 3).

### 2.3 Experimental recognition memory test

Table 4 demonstrates the performance of the frontal patients and controls on the nonverbal recognition test. Analysis of variance (ANOVA) was used to compare the performance of the two groups in terms of hits and correct rejections. The 2 (group) × 2 (response type) ANOVA revealed a significant main effect of group [*F*(1, 55) = 11.68; *p* < 0.01] where frontal patients performed significantly more poorly than controls. Indeed, the ROCs in Fig. 1 show that the frontal patients’ average ROC was well below that of the controls’ average ROC. There was not a main effect of response type (*p* = 0.06) or a significant group × response type interaction (*p* = 0.57).

**Fig. 3:**
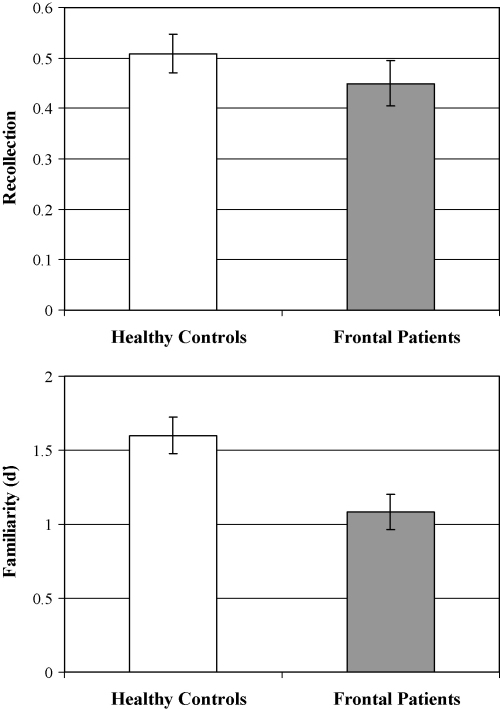
Estimates of recollection and familiarity derived using the dual-process ROC procedure for frontal patients and controls.

Fig. 3 shows the means and standard errors of the mean for recollection and familiarity estimates given by the dual-process model of Yonelinas et al. (1998). The frontal patients and controls did not differ significantly in terms of their recollection estimates (*p* = 0.32). Indeed, the ROC curves for both frontal patients and controls are clearly asymmetric along the diagonal, indicating the contribution of recollection processes. A second *t*-test comparing the familiarity estimates of the two groups revealed that frontal patients had significantly lower familiarity estimates than controls [*t*(55) = −2.92; *p* < 0.01]. Indeed, the frontal patients’ ROC for buildings was less curved than that of the controls, which indicates that familiarity is greatly reduced in the frontal group. The same results were found when negative estimates of recollection in younger and older controls were controlled for using the Cipolotti et al. (2006) method (see Bird et al., 2007; Cipolotti et al., 2006). In this case, recollection was estimated from the hits-false alarms of the first point on the function (hits-false alarm rates for six confidence responses only) instead of from the intersection of the regression line with the ordinate.

The frontal group was then subdivided into three anatomically defined subgroups according to the region of greatest damage (medial = 14, orbital = 5 and lateral = 5). Table 5 shows the means and standard deviations for the recollection and familiarity estimates of the subgroups of frontal patients. Separate one-way analyses of variance were conducted on the estimates data. For the recollection estimates, there was not a significant main effect of group (*p* = 0.16). However, for the familiarity estimates, there was a significant main effect of group [*F*(3, 53) = 3.96; *p* < 0.05]. The Dunnett one-tailed *t*-test was used to compare the familiarity estimates of each patient group against the healthy control group. The analysis revealed that both the medial and lateral groups had significantly lower familiarity estimates than the healthy controls (both *p* < 0.05). In contrast, the orbital group did not significantly differ from controls (*p* = 0.73).

A more detailed analysis of the stimuli was then conducted. Table 6 shows the frequency of each confidence rating (1–6) selected by the frontal patients and controls for targets and distractors. For the target stimuli, separate independent *t*-tests were conducted for each confidence rating (1–6) to compare the frequency that each one was selected by frontal patients and controls. To correct for multiple comparisons and to minimize the risk of type II errors, a Bonferroni correction was applied and only *p*-values ≤ 0.008 were considered statistically significant. The analysis revealed that none of these *t*-test comparisons were significant after Bonferroni correction (each *t* < 2.08). Therefore, in the case of target items, frontal patients and controls chose each confidence rating 1–6 a similar number of times.

For the distractors the same analysis was conducted. In this case, the only comparison that remained significant when a Bonferroni correction was applied was the *t*-test comparing the number of times confidence rating 5 was selected by frontal patients and controls [*t*(55) = 2.87; *p* = 0.006]. Frontal patients incorrectly chose confidence rating five significantly more often than healthy controls when rating distractor items.

A secondary analysis of the results was also conducted using a procedure appropriate for the unitary process models. Table 7 shows the slopes and sensitivity (da) parameters for the z-transformed ROC data of the frontal patients and controls. The slopes of both the frontal patients and the control group's z-ROCs did not differ significantly (*p* = 0.97), and were 0.7, which is within the range typically observed (Wixted & Stretch, 2004). Therefore, both groups had a similar variance factor. However, the analysis did reveal that the frontal patients demonstrated significantly lower sensitivity (da) i.e. how much more familiar the old items are compared to the new items (Macmillan & Creelman, 2003) than the controls [*t*(55) = −3.13; *p* < 0.005]. Therefore, memory strength (familiarity) was disrupted in the frontal patients but not the variance ratio.

### 2.4 General discussion

The aim of the present study was to examine further the effects of frontal lesions on recollection and familiarity. The experiment was designed within the context of the dual-process literature, within which the Yonelinas procedure was used to derive estimates of recollection and familiarity. We will initially consider the findings from the perspective of this methodology, as it allows direct comparisons to be made between the current results and other patient studies. However, clearly the data can also be considered from the perspective of the single-process theory.

The results show that patients with frontal lobe lesions have reduced values of familiarity-based recognition when estimates are derived using the ROC procedure. However, they do not have reduced recollection estimates. To our knowledge, this is the first group study to examine the effects of focal frontal lesions on the processes of recollection and familiarity using the ROC procedure (Yonelinas, 1994, 2002). The fact that there is a dissociation between impairments in recollection and familiarity has been taken as support for the dual-process theory of recognition memory.

Although the findings of Duarte et al. (2005) study fit with our finding that frontal lesions impair estimates of familiarity but not recollection, their reduced familiarity estimates derived using the remember/know procedure were specific to items encoded in the contra-lesional hemifield. Another way in which these two studies differ is in terms of the frontal regions that were primarily damaged. Duarte et al. (2005) involved a small sample of 9 patients who had incurred frontal damage due to infarction of the middle cerebral artery resulting in lesions centered in the dorsolateral and ventrolateral regions. In the current study, we included a larger sample of 24 patients whose aetiologies underlying their tissue damage were mainly tumour or haemorrhage of the anterior cerebral artery. Therefore, the majority of our patients had medial lesions, a large number of which also involved the lateral region. Indeed, studies that have conducted detailed anatomical analysis of their frontal patients’ lesions have revealed that memory retrieval impairments are associated with damage to both posterior medial regions and lateral regions (Alexander et al., 2003; Dimitrov et al., 1999; Turner, Cipolotti, Yousry, & Shallice, 2007). Therefore, it may be that the frontal patients in the current study were more severely impaired due to their additional medial involvement. However, this cannot be confirmed as Duarte et al. (2005) did not provide patients’ background scores on neuropsychological tests of executive abilities or memory.

When the frontal patients were subdivided into smaller groups depending upon which frontal region had the greatest damage, the analysis revealed that patients with predominantly lateral lesions and patients with largely medial lesions produced significantly lower familiarity estimates compared to healthy controls. However, patients with mainly orbital lesions did not differ significantly from controls. The reduction in familiarity estimates in these two subgroups of frontal patients is consistent with the recent neuroimaging studies that have reported activation associated with familiarity in both the lateral and medial frontal regions (e.g. Montaldi et al., 2006; Skinner & Fernandes, 2007; Vilberg & Rugg, 2007; Yonelinas et al., 2005). However, in conflict to inferences that might be tentatively drawn from the neuroimaging literature, neither the lateral nor the medial patient groups had impaired recollection estimates (Henson, Rugg, Shallice, Josephs, & Dolan, 1999; Skinner & Fernandes, 2007; Yonelinas et al., 2005). Moreover, when comparing patients with lateral and medial lesions on remember/know judgements, Wheeler and Stuss (2003) did not find that either patient group were impaired at know judgements. Thus, the literature on familiarity and recollection remains rather inconsistent.

However, it would be premature to draw too strong a conclusion about the mechanism responsible for the apparent familiarity deficit. A more detailed analysis of the number of times each confidence rating was selected by frontal patients and controls was also conducted. The analysis revealed that the frontal patients gave distractors a confidence rating of 5 significantly more often than controls. The two groups did not differ significantly in terms of the frequency that the remaining confidence ratings were selected for distractors (1–3, 4 and 6) or any of the target confidence ratings (1–6). As the frontal patients only differed significantly in terms of confidence rating 5 when rating distractors and not confidence rating 2 when rating targets, this suggests that their familiarity deficit may be attributed to their being unable to overcome the temptation to respond yes when lures seem familiar. Previous studies have suggested that the high false alarm rates in frontal patients performing recognition memory tests is due to the patients’ over dependency upon the general characteristics shared by targets and distractors and so distractors appear familiar (Curran et al., 1997; Delbecq-Derouesne et al., 1990; Schacter et al., 1996). Frontal patients may commit false alarms due to high similarity between distractors and stimuli included in the recognition task.

Some preliminary data which supports this view is provided by a similarity judgement control task where we asked independent raters to assess the similarity of the distractors which were frequently incorrectly rated 5 (or 6) by frontal patients, they compared with other distractor items which were correctly rated (1 or 2) by the patients in the recognition memory test. This study showed that those frequently incorrectly rated distractor items were rated by judges as being more similar to other items included in the experimental recognition memory test than the correctly rated distractor items. The findings suggest that the memory representations for the selected distractors have characteristics common with either one or more targets or other distractors. In this situation, any familiarity system by itself would be unable to guide accurate performance on the yes-no recognition task, as the distractor representations are not sufficiently distinctive and overlap with previously presented target and distractor representations. Instead, the recollection system would have to be called in. In support of this, there are several studies in the literature that have reported a deficit in distinguishing between targets and similar lures in patients with frontal lobe damage performing the Deese/Roediger-McDermott paradigm (e.g. Budson et al., 2002; Ciaramelli, Ghetti, Frattarelli, & Làdavas, 2006; Schacter et al., 1996). Therefore, the cause of the poor familiarity performance in frontal patients could relate to an inability to inhibit responses to close distractors or even potentially be a process related recollection rather than a general problem concerning familiarity, or a loss of a familiarity trace.

The other dominant approach to explain recognition memory, the single-process theory, assumes that one variable only influences recognition memory (e.g. Dunn, 2004; McClelland & Chappell, 1998; Shiffrin & Steyvers, 1997). Rather than memory retrieval occurring in an all-or-nothing manner, the UVSD theory states that memory retrieval is graded in strength on a single dimension ranging from no to limited to complete memory. In an attempt to assess whether the single- or dual-process theory of recognition memory best described the findings, the ROC data were also fitted to the UVSD model (e.g. Glanzer, Hilford, Kim, & Adams, 1999; Wixted, 2007). The shape of the recognition memory z-ROCs appeared linear rather than curvilinear, which is more consistent with the single- rather than the dual-process model (e.g. Glanzer et al., 1999). Although the application of signal detection theory to memory does not constrain how the slope of the z-ROC curve is interpreted, it has been suggested that it may reflect a recollection-like process (Aggleton et al., 2005). If this identification be accepted then it should be noted that the frontal patients did demonstrate significantly lower sensitivity (which would correspond to familiarity) than the controls. Therefore, the UVSD model could be held to lead to similar conclusions about the data as the dual-process theory with familiarity-type processes being impaired as a result of frontal lesions whereas the recollection-type (or variance ratio) processes remain intact.

It is however, thought that when recollection and familiarity contribute to performance on standard item recognition tasks, any curvilinearity of the z-ROC will only be subtle (e.g. Yonelinas, 1999b). The dual-process model proposes that the shape of the z-ROC depends on the role that recollection and familiarity play on item recognition performance. The greater the involvement of recollection, the more likely it is that the z-ROC will be u-shaped. In contrast, the greater the involvement of familiarity in recognition performance, the more linear the z-ROC will be (Yonelinas & Parks, 2007). The linear z-ROC in the current study would therefore need to be interpreted in terms of familiarity being more involved in performance. However, this is surprising given the high rate of 1 and 6 ratings in performance. On the dual-process model, one might have expected the z-ROCs to be more obviously u-shaped.

Nonetheless, the single- and dual-process views of recognition memory can be reconciled if recollection is thought of as a continuous process related to differing degrees of confidence. In that case, the memory strength variable is the additive combination of recollection and familiarity (Rotello, Macmillan, & Reeder, 2004; Wixted & Stretch, 2004; Wixted, 2007). Indeed, in a reply to Wixted (2007), Parks and Yonelinas (2007) point out that when they refer to recollection as being an “all-or-none” process, they mean that recollection can succeed or fail, not that everything or nothing about an item is recollected. Therefore, recollection can be graded.

Strikingly, whether the findings are interpreted in terms of either the single- and dual-process models, one has a double dissociation between the results of the current frontal patient group and those of patients with medial temporal lobe lesions. When the data were fit using the dual-process ROC method, our patients with frontal lobe lesions showed impaired familiarity and spared recollection estimates. This is in contrast to the more pronounced impaired recollection and spared familiarity estimates reported in patients with medial temporal lobe damage (see Yonelinas & Parks, 2007). Again, using the UVSD model to fit the data showed that frontal lobe damage disrupts memory strength, but has no affect on the variance factor. In contrast, medial temporal lobe damage disrupts variance ratio more than memory strength (Aggleton et al., 2005; Wais et al., 2006). These observed dissociations provide compelling evidence that prefrontal cortex and the medial temporal region play very different roles in memory.

In summary, analysis of the ROC data in the current study suggests that recollection and familiarity can be dissociated. While frontal patients’ recollection estimates remain intact, their familiarity estimates are significantly reduced compared to controls. It may be that this reduction is due to difficulty distinguishing between target and distractor items when they have a high degree of similarity. Therefore, frontal patients are more liberal in responding than controls and endorse more distractors. We suggest that future research contrasting the single- and dual-process theories of recognition memory should adopt tasks that rely more heavily on recollection such as source or associative recognition memory tasks.

## Figures and Tables

**Table 1:** Background information for frontal patients

Case	Age	Gender	Handedness	Aetiology	Time since surgery (months)
1	37	Male	Right	Glioma	1.0
2	45	Male	Right	Glioma	5.0
3	64	Male	Right	Glioma	2.0
4	40	Female	Right	SAH	38.0
5	34	Male	Right	Glioma	30.0
6	43	Male	Right	Glioma	2.0
7	32	Male	Right	Glioma	41.0
8	46	Male	Left	Glioma	6.0
9	43	Female	Right	Glioma	50.0
10	50	Female	Left	Glioma	N/A
11	58	Female	Right	Meningioma	2.0
12	17	Male	Right	TBI	8.0
13	64	Female	Right	Meningioma	1.0
14	68	Male	Right	Meningioma	38.0
15	31	Male	Left	ACoAA & SAH	2.0
16	62	Male	Right	Meningioma	11.0
17	48	Female	Right	Glioma	72.0
18	44	Male	Right	Glioma	35.0
19	27	Female	Right	ACoAA & SAH	1.0
20	73	Male	Right	Meningioma	4.0
21	60	Female	Right	ACoAA & SAH	25.0
22	31	Male	Left	Glioma	2.0
23	35	Female	Right	Glioma	N/A
24	27	Female	Left	Glioma	N/A

ACoAA = anterior communicating artery aneurysm; SAH = subarachnoid haemorrhage; TBI = traumatic brain injury.

**Table 2:** Mean age-scaled scores and standard deviations for the frontal patients and controls on the Doors and People test

	Frontal patients		Healthy controls	
	Mean	S.D.	Mean	S.D.
People test				
Immediate verbal recall	8.35	4.04	11.03	3.91*
Delayed verbal recall	9.70	2.48	11.03	1.70*

Names test				
Verbal recognition	10.78	3.49	12.66	3.06*

Shapes test				
Immediate visual recall	9.87	2.24	11.38	2.87*
Delayed visual recall	10.91	0.42	10.48	1.53

Doors test				
Visual recognition	8.57	3.09	10.38	3.05*

* *p* < 0.05.

**Table 3:** Performance of the frontal and control groups on the executive measures

	Frontal patients		Healthy controls	
	Mean	S.D.	Mean	S.D.
Verbal fluency (FAS) total words	32.46	16.47	52.73	12.66*
Stroop colour-word time (seconds)	154.40	53.22	125.23	27.76*
Trail Making test Part B (seconds)	85.22	49.55	69.27	19.98
Elevator test (max = 7)	6.38	1.25	6.97	0.17*
Proverbs test (max = 16)	5.79	3.15	7.91	2.27*

* *p* < 0.05.

**Table 4:** Frontal patients and controls’ mean performance (with standard deviations in parentheses) on the nonverbal recognition test

	Frontal patients		Healthy controls	
	Mean	S.D.	Mean	S.D.
Hits (max = 60)	47.42	7.43	52.30	4.96*
Correct rejections (max = 60)	45.96	6.46	49.58	6.11*

* *p* < 0.005.

**Table 5:** Estimates of recollection and familiarity for medial, lateral and orbital subgroups of frontal patients and controls

	Recollection		Familiarity	
	Mean	S.D.	Mean	S.D.
Medial (*n* = 1 4)	0.47	0.17	1.03*	0.54
Lateral (*n* = 5)	0.55	0.16	0.81*	0.64
Orbital (*n* = 5)	0.28	0.34	1.51	0.40
Healthy controls (*n* = 24)	0.51	0.22	1.60	0.72

* Dunnett one-tailed *t*-tests: medial and lateral groups significantly lower than healthy controls (*p* < 0.05).

**Table 6:** The slopes and sensitivity (Da) parameters for the z-transformed ROC data of the frontal patients and controls

	Frontal patients		Healthy controls	
	Mean	S.D.	Mean	S.D.
Slopes	0.67	0.14	0.66	0.20
Sensitivity (Da)	1.65	0.62	2.13	0.55*

* *p* < 0.005.

**Table 7:** The frequency of each confidence rating (1–6) selected by frontal patients and controls for targets and distractors

	Targets							Distractors				
	1	2	3	4	5	6	1	2	3	4	5	6
Frontal patients												
Mean	3.46	4.96	4.17	3.75	11.33	32.33	18.67	18.75	8.54	5.75	6.04*	2.25
S.D.	4.14	3.25	4.48	3.69	6.25	11.06	12.51	6.92	6.55	4.21	3.37	2.57

Healthy controls												
Mean	1.88	3.70	2.12	4.18	12.33	35.79	23.91	18.18	7.48	4.87	3.70*	1.85
S.D.	2.12	2.59	2.93	3.68	7.83	12.44	12.99	7.03	6.64	3.63	2.80	3.10

* *p* < 0.008.
